# Web-Based Cognitive Remediation Improves Supported Employment Outcomes in Severe Mental Illness: Randomized Controlled Trial

**DOI:** 10.2196/mental.6982

**Published:** 2017-09-20

**Authors:** Anthony WF Harris, Tanya Kosic, Jean Xu, Chris Walker, William Gye, Antoinette Redoblado Hodge

**Affiliations:** ^1^ Westmead Institute for Medical Research Brain Dynamics Centre University of Sydney Westmead Australia; ^2^ Discipline of Psychiatry University of Sydney Westmead Australia; ^3^ Department of Psychology Macquarie University Sydney Australia; ^4^ Sunflower Health Services Schizophrenia Fellowship of New South Wales Gladesville Australia; ^5^ Recovery Services Schizophrenia Fellowship of New South Wales Gladesville Australia; ^6^ Child Development Unit New South Wales Centre for Effective Reading Children's Hospital at Westmead Westmead Australia

**Keywords:** severe mental disorders, supported employment, cognitive function, cognitive remediation, randomized controlled trial

## Abstract

**Background:**

Finding work is a top priority for most people; however, this goal remains out of reach for the majority of individuals with a severe mental illness (SMI) who remain on benefits or are unemployed. Supported employment (SE) programs aimed at returning people with a severe mental illness to work are successful; however, they still leave a significant number of people with severe mental illness unemployed. Cognitive deficits are commonly found in SMI and are a powerful predictor of poor outcome. Fortunately, these deficits are amenable to treatment with cognitive remediation therapy (CRT) that significantly improves cognition in SMI. CRT combined with SE significantly increases the likelihood of individuals with severe mental illness obtaining and staying in work. However, the availability of CRT is limited in many settings.

**Objective:**

The aim of this study was to examine whether Web-based CRT combined with a SE program can improve the rate return to work of people with severe mental illness.

**Methods:**

A total of 86 people with severe mental illness (mean age 39.6 years; male: n=55) who were unemployed and who had joined a SE program were randomized to either a Web-based CRT program (CogRem) or an Internet-based control condition (WebInfo). Primary outcome measured was hours worked over 6 months post treatment.

**Results:**

At 6 months, those participants randomized to CogRem had worked significantly more hours (*P*=.01) and had earned significantly more money (*P*=.03) than those participants randomized to the WebInfo control condition. No change was observed in cognition.

**Conclusions:**

This study corroborates other work that has found a synergistic effect of combining CRT with a SE program and extends this to the use of Web-based CRT. The lack of any improvement in cognition obscures the mechanism by which an improved wage outcome for participants randomized to the active treatment was achieved. However, the study substantially lowers the barrier to the deployment of CRT with other psychosocial interventions for severe mental illness.

**Trial Registration:**

Australian and New Zealand Clinical Trials Registry (ANZCTR) 12611000849998; http://www.anzctr.org.au/TrialSearch.aspx?searchTxt=12611000849998&isBasic=True (Archived by WebCite at http://www.webcitation.org/6sMKwpeos)

## Introduction

Functional recovery in people with a severe mental illness such as schizophrenia remains poor with high rates of dependence upon government benefits and significant difficulty with social isolation. An important index of a good recovery is a return to employment as this requires the individual to be able to combine motivation, cognitive performance, and the ability to relate to others. However, in the developed world few people with schizophrenia are employed. In the clinical antipsychotic trials of intervention effectiveness (CATIE) study, only 14.5% of subjects with schizophrenia had participated in competitive employment in the month before enrollment in the study [1]. In Europe, in a survey of the United Kingdom, France, and Germany by Marwaha and colleagues, only 7.6-11.8% of people with schizophrenia were supporting themselves entirely through work [2]. In Australia, only 22.4% of people with a psychotic disorder were in either part or full time employment, and this employment rate had not changed in a decade [3] despite paid employment being a priority for many [4]. This failure to return to competitive employment ensures continued poverty and marginalization for most people with a severe mental illness and shuts them out of important sources of socialization and integration with the rest of the community.

The reasons for such poor rates of employment are numerous and include the obvious effects of illness, especially negative symptoms [1,5] and the interruption to education and training caused by the onset of a psychotic illness during late adolescence and early adulthood [6,7]. However, one of the most significant contributors to poor outcome are the neurocognitive deficits of psychosis [1,8-10]. These deficits are broad based and severe [11], exist at the time of first presentation to mental health services [12], and persist [13]. Unfortunately existing pharmacological approaches fail to treat these deficits [14]. However, the development of effective treatments for cognitive deficits in psychosis variously known as cognitive remediation therapy (CRT) or cognitive training, suggests an alternate treatment approach to these problems [15,16]. Importantly, this translates into improved real life functioning more readily if CRT is combined with another psychosocial intervention [15,16]. One such intervention that has been shown to consistently improve return to employment in people with a severe mental illness is supported employment [17].

A number of trials have examined the combination of cognitive remediation with employment-based interventions [18-25]. Bell and colleagues combined CRT with a transitional employment program to significantly increase hours of work over 6 months [20]. In a second study over 24 months, individuals who received both CRT and a vocational intervention worked more hours and were more likely to stay in work than those who received the vocational intervention alone [19]. These gains were best in the participants with the lowest community functioning [25]. McGurk and colleagues also demonstrated that the addition of CRT to vocational programs significantly improved the likelihood of successful placement in and retention of employment for individuals with schizophrenia [18,21]. On the other hand, Au and colleagues [23] were unable to find an advantage for combining CRT with supported employment in Hong Kong in a trial notable for its high rate of job placement.

The extensive use of computer-based cognitive remediation in treatment raises the question of whether Internet-delivered cognitive remediation without the extensive use of skilled therapists is effective. This is a rapidly expanding part of Internet-delivered services; however, a recent review has cast doubt on provider’s claims of effectiveness [26]. Nonetheless, the development of Internet-based CRT provides a means of delivering treatment in a way that enables a far larger number of people with cognitive difficulties to engage with it.

The aim of this study was to combine a supported employment (SE) program with Internet-based CRT in a randomized controlled trial (RCT) to test if this could improve the employment outcomes for people with a severe mental illness in frontline services.

## Methods

### Study Design

This study is an RCT of Internet-based cognitive remediation plus supported employment (CogRem) versus Internet-based information plus supported employment (WebInfo). In total, 89 participants were recruited from supported employments services situated in metropolitan and regional New South Wales, Australia. All participants who were unemployed and actively seeking work via a SE program (the Disability Employment Service [DES]) were invited to take part in the study. Participants were in the age range of 17-65 years, had English language skills adequate for understanding written instructions and completing assessments, and had a diagnosis of a severe mental illness (schizophrenia, schizophreniform disorder, schizoaffective disorder, bipolar disorder, or psychotic depression). Exclusion criteria were limited to having an intellectual disability or a diagnosis of substance dependence other than nicotine or caffeine. All sites were rated as to their compliance with the SE model using the Supported Employment Fidelity Scale [27].

Prospective participants were invited to take part in the study by their DES case manager. After reading a participant information sheet and agreeing to it, they logged on to a purpose built website and were asked to complete demographic and baseline measures. After the completion of those measures, they were randomized to one of two Internet-based programs and asked to log on twice weekly to that website either at home, their DES office, or at another rehabilitation support site. All participants had access to computers either at their DES provider or at a Clubhouse at a minimum. DES case managers were asked to encourage participants to continue to log on but were not expected to provide any other coaching or intervention. They were not blind to the allocation of the participant. The DES case managers recorded employment outcomes in detail as the performance on these outcomes form the basis of remuneration to the program by the federal government. This information was recorded for the 6 months after employment commences and was independently audited for accuracy by external government agencies.

### Measures

Assessment of cognition was carried out before randomization and at 6 months using the WebNeuro, which is an Internet-based neuropsychological battery [28]. The cognitive domains tested included attention and concentration (continuing performance task—reaction time, omission, and commission error rates), working memory (digit span forward and trials correct), memory recognition (word list recognition and learning rate), information processing speed (verbal interference, choice reaction time, and switching of attention), response speed (motor tapping), and executive functioning (maze completion time and total errors). The battery takes 45 min to complete, and there are multiple forms for repeated assessment [29]. Scoring was conducted using automated software embedded in the program, and data were downloaded from the Brain Resource website.

DES staff collected information on hours worked, wages, number of jobs, and type of jobs at 6 months. This was required by the government agency funding the SE package. Other outcomes included paid work, voluntary work, or education. Function was also measured using the Role Functioning Scale [30], an observer rated scale with four domains: working productivity, independent living or self-care, immediate social network, and extended social network. This was also completed by the DES worker.

Symptomatology was measured using the Behavior and Symptom Identification Scale-24 (BASIS-24) [31], a self-rated scale measuring a broad range of psychopathology and substance use developed and validated for Web use. Function and quality of life was self-rated using the World Health Organization Quality of Life-BREF (WHOQOL-BREF), a 26-item rating scale [32]. These ratings were completed on the Web via the CogRem portal.

### Intervention

Participants accessed all material via a purpose built website. This website was used to centralize all assessments, except for the cognitive testing that required a separate log-on to another website. Once randomized, participants were provided with password access to either the treatment (CogRem) or control group (WebInfo) and were sent an email with instructions. This information was also sent to the DES workers so they could assist participants if required. Participants in the CogRem group were requested to use 4 commercially available cognitive training packages—Lumosity, Brain HQ, MyBrainSolutions, and Scientific Brain Training Pro. Participants were not directed to any one exercise or website but suggested to sample and use as many as they liked. All access and costs for this were paid over a 4-month period. Access to BrainHQ was introduced after that product became available; access to Scientific Brain Training Pro ceased after the shutting down of that service. The control participants (WebInfo) were able to log on through the project portal to a large number of free news (eg, ABC News), information (weather and public transport planner), and entertainment (eg, YouTube and music) websites, none of which contained games used with cognitive remediation.

All participants were asked to log on through the study portal twice a week for a total of at least ten hours over a 4-month period. Adherence was monitored by the research team who also provided regular reminders to participants to log on via email. Time on the study website and choice of website was recorded; however, time on proprietary sites beyond the entry onto the study portal, that is, time on a particular game, could not be monitored.

Participants were welcomed into the study and contacted by both telephone and email to encourage them to continue in the study. Participants also received birthday and Christmas cards. There was no direct face-to-face contact with study staff; however, the participants continued to have regular visits with their SE worker. All participants received Aus $25 gift voucher following the completion of the 10-hour training and a further Aus $25 gift vouchers for the completion of the follow-up assessment measures at 6 and 12 months. DES workers and research team provided assistance to participants as required. All DES workers were given a brief orientation to the trial and were encouraged to refer their clients to the study.

### Randomization

An independent statistician generated the randomization sequence using SPSS version 15 (IBM Corp). Patients were randomly allocated to either CogRem or WebInfo in blocks of 8 with a 1:1 allocation ratio. This method ensures that the treatment sample sizes were equal after every batch of 8 enrollments. The sequence generated was placed in order in sealed opaque envelopes by an independent person and were opened at the time of allocation by the research assistant. Allocation was in order of contact via the website and was concealed from the participants and their case workers who could only access the study via the website. The study coordinator had no prior knowledge of any participant at the time of allocation and was blind as to the allocation until the envelope was opened. Participants and DES workers were not blind to allocation nor was the research assistant coordinating the project.

### Ethics

All participants in this study received written information about the project, and written consent was obtained. The study was approved by the University of Sydney Human Research Ethics Committee (project no. 2012/1350). The trial was registered with the Australian and New Zealand Clinical Trial Registry no: 12611000849998 before any participant recruitment.

### Statistical Analysis

The sample size calculation (n=150) was based upon published effect sizes of combined cognitive remediation and psychosocial interventions [16]. This effect size was then significantly discounted by half to better reflect the Web-based delivery of the package. A dropout rate of one-third was factored into the final subject numbers [33]. Demographic characteristics were tested using *t*-tests and chi-square tests. The primary outcome for the study was wages earned. Secondary outcomes included number of hours worked, number of days worked, number of hours paid, and number of jobs. These outcomes were tested using nonparametric statistics due to the distribution of results. Secondary outcomes were corrected for multiple comparisons using a Bonferroni correction. Neuropsychological test results were compared using an analysis of variance (ANOVA) design. All statistics were performed using SPSS version 21.0.

## Results

### Participants

**Figure 1:**
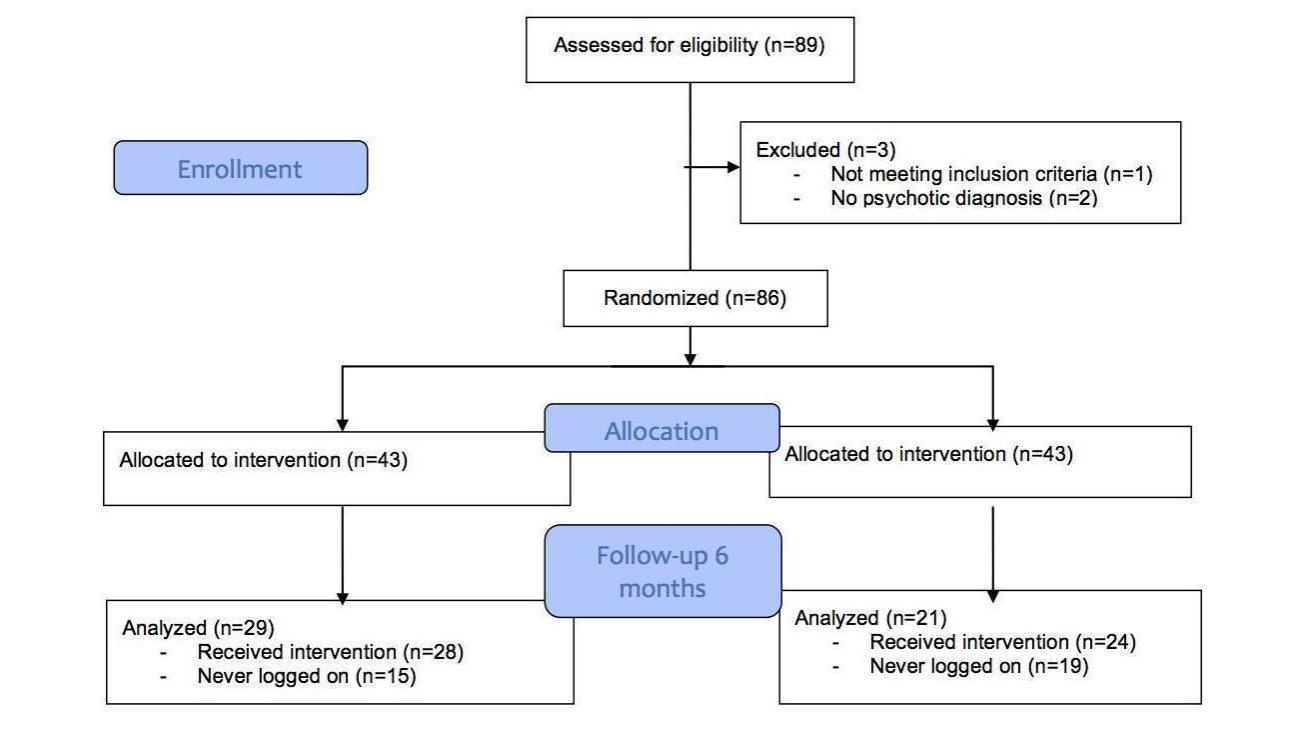
Consolidated Standards of Reporting Trials (CONSORT) diagram.

In total, 89 participants were enrolled into the study with 3 being excluded before randomization as they either did not have a psychotic disorder (n=2) or already had a job (n=1). Of the 86 participants who continued, 43 were randomized to each of the two experimental arms—CogRem or WebInfo (see Figure 1). The two groups were well balanced on gender, diagnosis, years of education, medication dose, psychopathology, and baseline functioning (see Table 1). Levels of symptomatology were consistent with other samples of community mental health participants [31]. However, the CogRem group was significantly older than the WebInfo group 42.3 years (standard deviation [SD] 11.0) versus 36.8 years (SD 10.7; df=83; *P*=.02).

Participants were recruited from 8 different sites. The number of participants from each site varied from 2 to 32 with the majority coming from 2 sites. Randomization remained satisfactory across all sites. Sites were assessed using the Supported Employment Fidelity Scale [27] and scored an average of 96.9 (SD 8.2) with a range from 84-107 on this scale, indicating fair to good adherence to the supported employment model.

**Table 1:** Demographics of participants at baseline.

Variable		CogRem (n=43)		WebInfo (n=43)		Difference
Gender: male, n (%)		25 (58)	30 (70)		*P*=.37^a^	
Age (years), mean (SD^b^)		42.3 (11)	36.8 (10.7)		*P*=.02	
**Diagnosis (n)**					*P*=.53	
	Schizophrenia	28	22			
	Schizoaffective	2	3			
	Bipolar	11	16			
	Other psychotic	2	2			
**Education (n)**					*P*=.56	
	≤ year 10	11	11			
	Year 12	11	11			
	Trade or other	11	14			
	University	9	5			
CPZ^c^ equivalent, mean (SD)		256 (287)	276 (342)		*P*=.79	
BASIS-24^d^ total score, mean (SD)		1.52 (0.66)	1.66 (0.79)		*P*=.56	
Role Functioning Scale, mean (SD)		22.7 (3.7)	22.8 (3.5)		*P*=.91	

^a^NS: not significant.

^b^SD: standard deviation.

^c^CPZ: chlorpromazine.

^d^BASIS-24: Behavior and Symptom Identification Scale-24.

### Participation and Engagement in Study

Dropout was high across the trial with only 49 subjects (57.0%, 49/86) being followed up at 6 months. This did not differ significantly between arms of the study. Subjects who dropped out usually never logged on to the website and were not exposed to either the CogRem (15/43, 35%) or to the control WebInfo (19/43; 44%) site. There was no significant difference in gender, age, diagnostic group, medication dose, or premorbid education between those who dropped out or continued in the trial. However, there was a significant difference on two subscales of the Role Functioning Scale with the participants who dropped out less likely to have a good immediate (*t*_82_=3.37, *P*=.001) or extended (*t*_82_=2.754, *P*=.007) social network. Those who did continue with the study were exposed to an equivalent amount of content from the study website (CogRem: median [Md]=5.5 hours, WebInfo: Md=6.9 hours; Mann Whitney *U*=331.5, *P*=.93). Those who remained in the study were engaged with their SE case managers seeing them a median of 25 times over the 6-month period.

### Employment Outcomes

A total of 23 participants returned to some work during the 6-month follow-up, though for the majority it was infrequent. On the primary outcome for the study (see Table 2) of work place involvement, the CogRem group (Md=168, n=27) worked a greater number of hours than the WebInfo group (Md=50, n=19) (*U*=143.5, *P*=.01), more of which were paid (CogRem: Md=100, n=29; WebInfo: Md=0, n=21) (*U*=202.5, *P*=.04) and earned significantly more wages. Of wages earned, there was a significant difference between the groups with the CogRem group (Md=Aus $1950, n=29) earning significantly more money than the WebInfo group (Md=Aus $0, n=20) (*U*=189.5, *P*=.03). This pattern was repeated across measures and a greater number of paid hours. However, these two results were no longer significant after correction for multiple comparison; the latter two results referring to paid work as against paid and voluntary work or formal study. The two groups did not differ significantly in the number of paid jobs found over the 6 months (CogRem: Md=1, n=29; WebInfo: Md=0, n=20) or the number of days worked (CogRem: Md=51, n=24; WebInfo: Md=20, n=17). There were no significant differences between the two groups in regards to symptomatology or quality of life. There were no differences in relapse rates between the two groups.

Despite the significant differences in financial outcome, there were no significant change in neurocognition with the Internet-based cognitive remediation intervention in any of the cognitive domains between the two groups. There was no change psychopathology as measured on the BASIS-24 or on quality of life.

**Table 2:** Results.

Variable	CogRem		WebInfo		Difference
	Average or Md^a^	SD^b^ or range	Average or Md	SD or range	
No. contacts DES^c^	33	29	33	31	*P*=.99^d^
Hours on the Web^a^	5.5	0-21	6.9	0-19	*P*=.42
Hours worked^a^	180	0-1040	50	0-312	*P*=.01; *U*=143.5
No. days worked^a^	51	0-130	20	0-130	*P*=.17
No. of jobs	1	0-2	0	0-1	*P*=.27
Hours paid^a^	156	0-1040	50	0-312	*P*=.05; *U*=89.5
Total money earned^a^	Aus $1950 (US $1562)	Aus $0-31200 (US $0-24989)	0	Aus $0-6408 (US $0-5132)	*P*=.03; *U*=189.5
Role Functioning Scale	23.0	3.8	22.1	4.1	*P*=.41

^a^Md: median.

^b^SD: standard deviation.

^c^DES: Disability Employment Service.

^d^NS: not significant.

## Discussion

### Principal Findings

This trial supports the advantages of combining cognitive remediation therapy with supported employment in people with a severe mental illness who wish to return to work [18,21,24]. It extends this area of research by demonstrating that CRT could potentially be delivered via the Internet, considerably broadening the number of services that could provide combinations of CRT with supported employment. If this was replicated, it would decrease the dependence upon specially trained mental health professionals to deliver the therapy which has limited its provision to specialist services. This trial suggests that the range of settings in which CRT could be provided can be increased while maintaining at least some of the effectiveness of the therapy.

### Why Did the Intervention Work?

The improvement in employment outcomes was found without significant changes being observed in cognition. Although other studies have observed changes in function with minimal or no neurocognitive improvement with CRT [34-37], the result begs the question of the mechanism of effect. Also, participants experienced relatively small doses of CRT. Whereas participants were asked to complete 10 hours on the Web, the median exposure was 5.5 hours. It is unclear what the necessary “dose” of CRT is, and it is possible that at least some of the benefits of CRT in engaging participants in thinking skills may be found after short courses of CRT. Wykes and colleagues did not find a significant effect for duration of treatment on function in their meta-analysis of CRT [15], yet found a moderate effect for CRT on functioning.

It might be argued that the Web-based intervention provided in this trial was not CRT and could not be expected to have the same effect as CRT on cognition. Certainly, the total period of exposure to the intervention and the intensity of exposure was low compared with other studies of CRT; however, the decision to choose commercially available educational cognitive exercises was based upon the Neuropsychological Educational Approach to Cognitive Remediation (NEAR) [38], a technique that has successfully integrated a broad range of software into CRT. As we were unable to access the detail of game choice and performance, we are unable to report on a more granular analysis of training, cognitive improvement, and functional outcome. This would be one of the targets of future work.

The lack of any significant change in cognition might be as a result of the Web-based neurocognitive measures used. These tests may not have the same sensitivity to change as observed in face-to-face testing nor have been performed in a consistent or rigorous fashion resulting in a considerable variation of results. Motivation to engage in neuropsychological testing without a trained administrator to encourage and assist a person is also suspect.

Participants did not show significant changes in their levels of psychopathology as measured by the BASIS-24. This is consistent with other studies that have seen few changes in psychopathology during treatment with CRT [15]. Little movement was seen in the scores elicited on the Role Functioning Scale. This may have been due to a reliance on DES case managers who had little familiarity with the use of such scales. Future work would be strengthened by face-to-face assessments by a researcher blind to participant allocation or the use of scales that did not require expert mental health worker input.

### Was the Chosen Outcome Valid?

Hours in work, rather than change in neurocognition, was the primary outcome in this trial. There were three main reasons for this. First, return to open employment is a priority for people with a severe mental illness [39]. Employment has a real potential to help those individuals break through into the wider community and lift at least some people with a severe mental illness out of poverty. The amount of money earned has a clear and definite value. Second, the hours of work and amount of money earned by the person could be accurately determined. Participation in work is the basis for reimbursement by the Australian government to the SE program, and wages earned is a key performance indicator that is tightly measured and independently audited. Successful placement in work forms the basis of ongoing contracts for those services. Hence, they were likely to be the most accurately measured by non-mental health staff. Evidence such as pay slips were required to prove that individuals had returned to paid employment; hence, we are confident that the payments recorded accurately reflect what individuals earned. Finally, the participants were widely dispersed over a large geographical area and were being seen by supported employment workers with no research or specialist mental health skills. During planning for the study, it was thought impractical to train DES staff to be accurate and reliable raters of mental health measures, partly due to a lack of basic training in psychopathology and research method and partly in recognition of the high staff turnover in these positions. This also influenced the choice of Web-based measures of neurocognition and self-rated scales of psychopathology.

### Limitations

The trial was significantly affected by operational issues in the supported employment provider which reduced recruitment. These difficulties amplified the lack of contact with the research team. DES staff and participants had little interaction with study staff beyond receiving emails and calls encouraging continued involvement in the study. This reliance on non-health or research staff may have been one of the factors responsible for the high dropout of participants from the program (43%). Importantly, subjects who dropped out did not differ from those who continued on gender, age, or premorbid education; however, they were already less socially integrated with family or their community. Other studies have suffered lower rates of dropout in the United States [18,19,40] or Hong Kong [23]; however, these studies had face-to-face input by mental health professionals. Studies of Web-based health interventions have observed similar rates of dropout [33].

No change was seen in the neurocognition test results. This may have been because of there was no change of scores with the CRT provided; however, it may also reflect poor motivation in those who interacted with the Web-based neurocognitive battery. The battery does appear to have adequate psychometric properties to detect any change [28]. A further possibility is that participants just simply did not interact with the training. Measurement of activity on the website is necessary before a better understanding of the mechanism of action of the treatments. This will be a target of future research.

The study was well controlled with no significant difference being observed between arms in level of education, study discontinuation, uptake of the CogRem website, or contact with a DES case manager. The active control successfully engaged participants randomized to it with no difference in overall hours spent in either arm of the study. Similarly, the intensity of DES case manager involvement was high in both arms of the study, underlining that differing levels of DES worker support was not the reason for the superiority of the CogRem intervention. The number of hours of DES worker contact also suggests that an active model of supported employment was being applied across sites. The arms did differ in the average age of participant; however, it was the WebInfo arm that was on average younger (36.8 years vs 42.3 years), biasing the study if anything to the active control arm of the study.

### Conclusions

This study supports the value of combining psychosocial treatment such as Web-based CRT with supported employment services for people with a severe mental illness. Further work is planned to enhance Web-based neurocognitive CRT with social cognitive remediation as the immediate effects of social cognition upon functional outcome is possibly far greater than that of neurocognition [10,41].

## Appendix

Multimedia Appendix 1 CONSORT-EHEALTH checklist (v1.6.1).

## Abbreviations

ANOVA: analysis of variance
BASIS-24: Behavior and Symptom Identification Scale-24
CATIE: Clinical Antipsychotic Trials of Intervention Effectiveness
CRT: cognitive remediation therapy
DES: Disability Employment Service
NEAR: Neuropsychological Educational Approach to Cognitive Remediation
RCT: randomized controlled trial
SD: standard deviation
SE: Supported Employment
WHOQOL-BREF: World Health Organization Quality of Life-BREF
